# Recurrent fifth metatarsal stress fractures in a professional soccer player with hypoparathyroidism: a case report

**DOI:** 10.1186/s12891-020-03383-2

**Published:** 2020-06-03

**Authors:** Itaru Kawashima, Atsushi Yamaga, Ryosuke Kawai, Yushi Hoshino, Shinya Ishizuka

**Affiliations:** 1 Department of Orthopaedic Surgery, Nagoya University Graduate School of Medicine, 65 Tsurumai, Showa-ku, Nagoya, Aichi 466-8550 Japan; 2 Department of Orthopaedic Surgery, Asahi University Hospital, 3-23 Hashimotocho, Gifu, 500-8523 Japan; 3 Department of Orthopaedic Surgery, Yamaga Orthopaedic Clinic, 9-15 Masagocho, Gifu, 500-8864 Japan

**Keywords:** Fifth metatarsal stress fracture, Hypoparathyroidism, Hypocalcemia, Soccer, Case report

## Abstract

**Background:**

Hypoparathyroidism is characterized by low or inappropriately normal levels of parathyroid hormone leading to hypocalcemia. In this report, a case of recurrent fifth metatarsal stress fractures in a professional soccer player with hypoparathyroidism is presented.

**Case presentation:**

A 23-year-old male professional soccer player developed left foot pain. He had no specific medical or family history. He was diagnosed with a fifth metatarsal stress fracture and underwent osteosynthesis with a cannulated cancellous screw 3 days after the injury. After three and a half months, the X-ray showed bone union, and he returned to full sports activity. However, he felt pain in his left foot again, and a re-fracture was found on X-ray a week later. Osteosynthesis was performed again. Two months after re-operation, the cause of re-fracture was investigated. Laboratory results showed abnormally low levels of serum calcium (8.4 mg/dL) and intact parathyroid hormone (i-PTH: 19.0 pg/mL). However, other laboratory examinations were normal. Therefore, he was diagnosed with primary hypoparathyroidism according to the diagnostic criteria. Medical treatment was started with alfacalcidol 1.0 μg/day. One month after starting medication, the serum calcium improved to 9.4 mg/dL. Four months after the re-operation, the X-ray showed bone union, and he was therefore allowed to play soccer. While he played professional soccer, there were no new subjective complaints.

**Conclusions:**

Hypoparathyroidism may be one of the risk factors for stress fractures. We believe that serum calcium levels should be checked in patients with stress fractures, and if the serum calcium is low, hypoparathyroidism should be considered.

## Background

Hypoparathyroidism is a rare metabolic disorder characterized by low or inappropriately normal levels of parathyroid hormone leading to hypocalcemia [1]. On the other hand, stress fracture of the fifth metatarsal bone is well described in the literature [3, 4, 7, 8, 17]. However, re-fractures after fixation with a screw and completed healing have been mentioned significantly less often in previous studies [10].

A previous study showed that the prevalence of fragility fractures is greater in patients with hypoparathyroidism [5]. However, to the best of our knowledge, there are no previous reports of stress fractures with hypoparathyroidism. In this report, the case of a professional soccer player who had been twice treated surgically for left fifth metatarsal stress fractures and was diagnosed with hypoparathyroidism after re-fracture surgery is presented.

## Case presentation

A 23-year-old male professional soccer player visited our hospital because of left foot pain. He had had slight pain in his left foot while playing soccer 1 month before the visit and noticed severe pain a day before the visit, with no history of any injury or trauma. His height, weight, and BMI were 181 cm, 77 kg, and 23.5 kg/m^2^, respectively. He had no specific medical or family history. He had never smoked. He had never experienced paresthesiae, tetany, or convulsions. X-ray and computed tomography (CT) examinations showed a fifth metatarsal stress fracture. Three days after the onset of acute severe pain in his left foot, osteosynthesis with a cannulated cancellous screw with a diameter of 4.5 mm in the fifth metatarsal bone was performed.

He started jogging after two and a half months. After three and a half months, X-ray examination showed bone union, and he returned to play soccer without an orthosis. However, the patient felt pain and discomfort in his left foot with a sense of insecurity a week later. Three months and 3 weeks after the first operation, X-ray examination showed re-fracture of his left fifth metatarsal, despite the screw fixation remaining in situ. Osteosynthesis was again performed, this time with a thicker headless compression screw. After the second operation, his ankle was immobilized with a brace for 2 weeks.

**Fig. 1:**
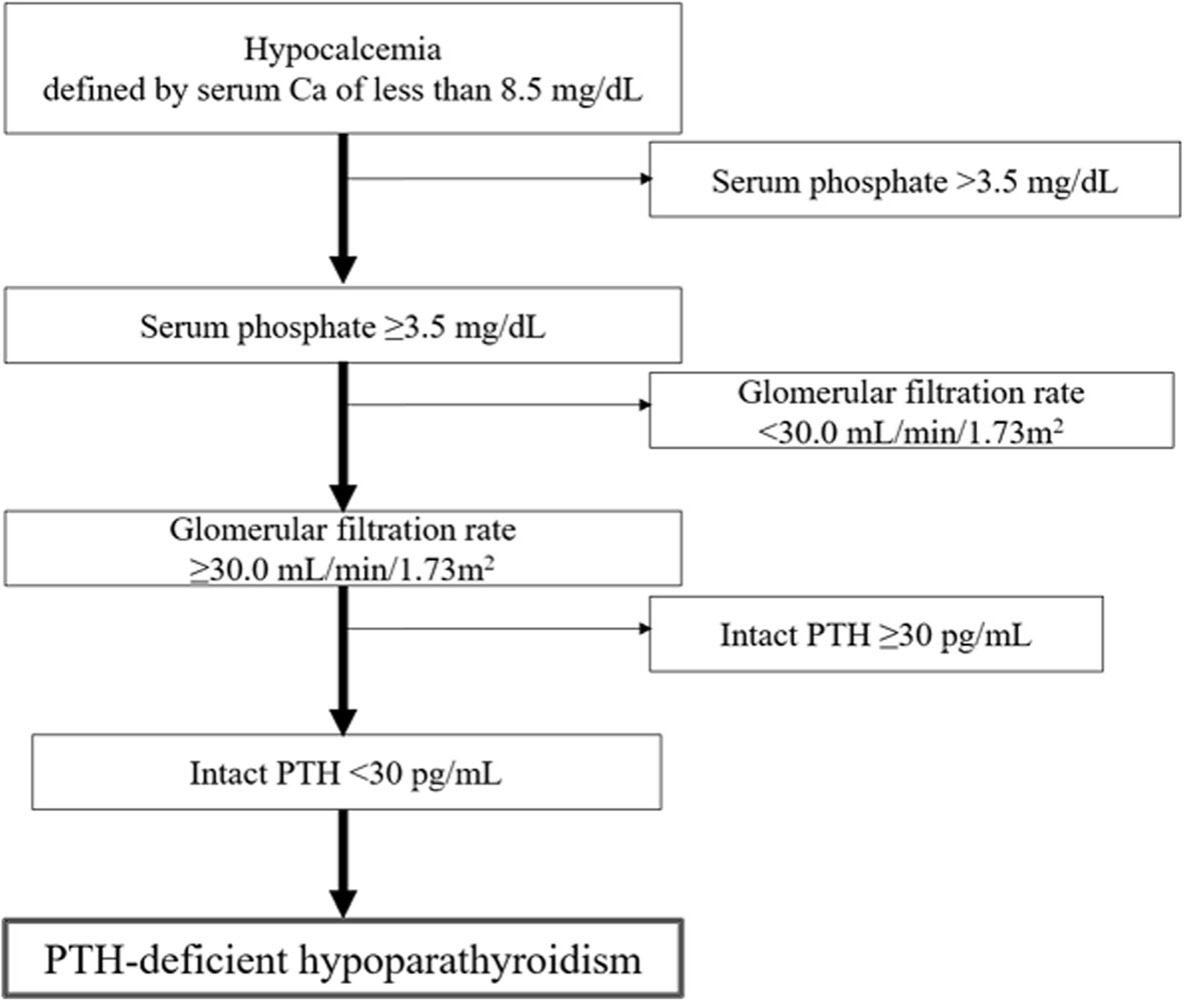
Algorithm for evaluating patients with hypocalcemia and hypoparathyroidism. Figure based on information in Fukumoto et al.^9^

Two months after re-operation, the cause of the re-fracture was investigated. Laboratory results showed abnormally low levels of serum calcium (8.4 mg/dL, normal range 8.6–10.2 mg/dL) and intact parathyroid hormone (i-PTH: 19.0 pg/mL, 10–65 pg/mL). Serum 25(OH) D was slightly low (22 ng/mL). However, other laboratory examinations, including serum phosphorus (4.2 mg/dL, 2.5–4.5 mg/dL), serum magnesium (1.8 mg/dL, 1.2–2.2 mg/dL), serum alkaline phosphatase (161 U/L, 50–340 U/L), serum creatinine (0.91 mg/dL, 0.61–1.04 mg/dL), eGFR (89.8 mL/min/1.73 m^2^, > 60 mL/min/1.73 m^2^), serum 1–25(OH)_2_D (42.5 pg/mL, 20–60 pg/mL), free T3 (3.27 pg/mL, 2.3–4.3 pg/mL), and free T4 (1.39 ng/dL, 0.9–1.7 ng/dL), were normal (Table 1). Bone mineral density (BMD) was very high using dual X-ray technology at L2, L3, L4 (1.474 g/cm^2^, T-score 2.4) and the total hip (1.698 g/cm^2^, T-score 5.8). CT of the brain and neck was normal. The patient was diagnosed with primary hypoparathyroidism according to the diagnostic criteria, given the absence of other manifestations of endocrinopathy, acquired causative factors such as surgery, radiation therapy, infection, or any family history [9] (Fig. 1).

**Table 1:**
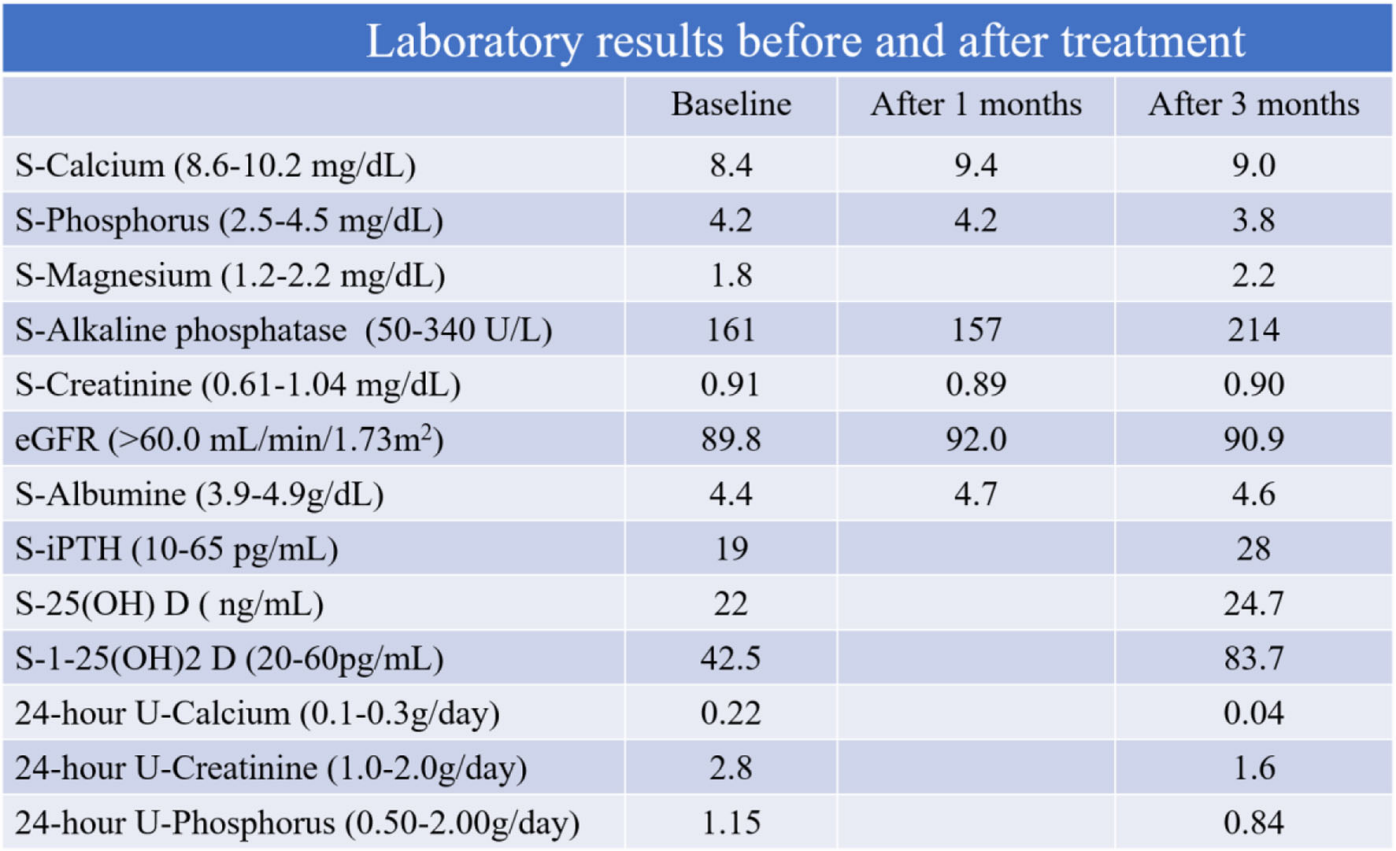
Patient’s laboratory results

**Fig. 2:**
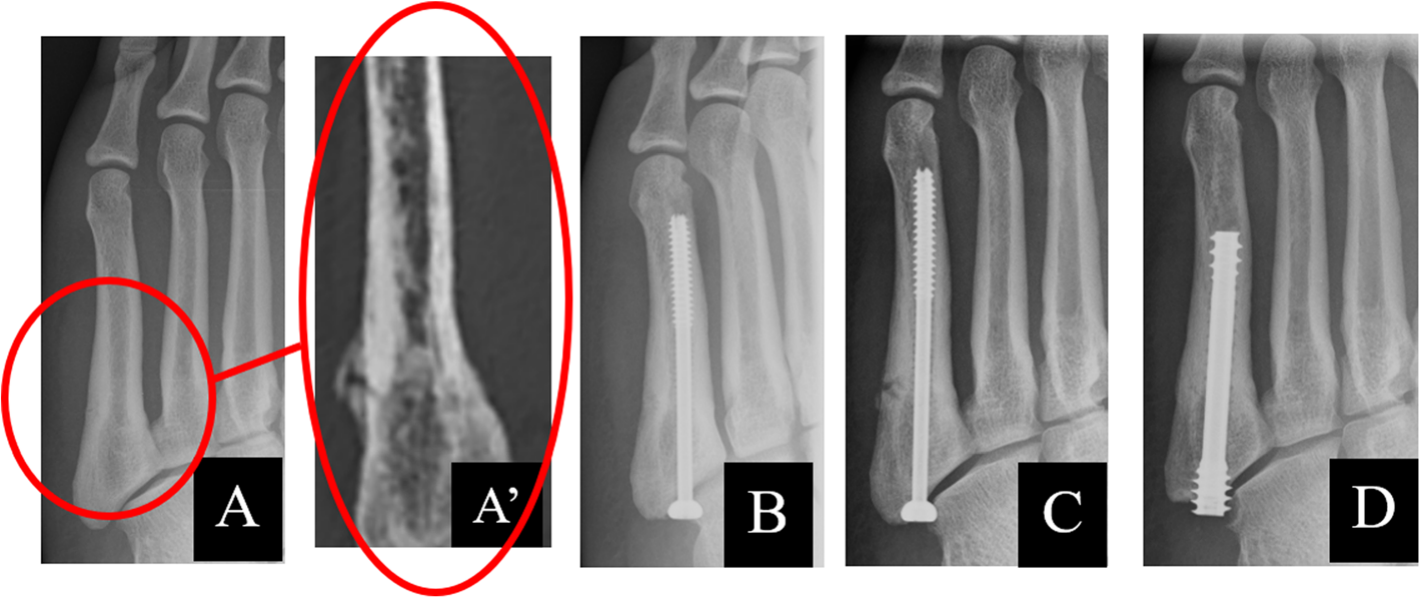
**a** Radiographs of the left foot before the primary operation. (A’) CT scan of the left foot before the primary operation. **b** Radiographs two months after the primary operation with a 4.5-mm-diameter screw. **c** Radiographs showing the re-fracture 3.5 months after the primary operation. **d** Radiographs showing bone union 3 months after re-operation

Medical treatment started with oral administration of alfacalcidol 1.0 μg/day. One month after the start of treatment, the serum calcium improved to 9.4 mg/dL. X-ray examination showed bone union, and he was allowed to run (Fig. 2).

Four months after re-operation, the X-ray showed visible complete remodeling of the location of the re-fracture of the base of the fifth metatarsal bone, and he was therefore allowed to play soccer. Five months after re-operation, laboratory results showed normal levels of serum calcium (9.0 mg/dL), i-PTH (25.0 pg/mL), serum 25(OH) D (30 ng/mL), serum phosphorus (3.8 mg/dL), and serum magnesium (2.2 mg/dL). While he played professional soccer, there were no new subjective complaints related to the operated foot, and the X-ray of the foot taken at the last check was normal, with visible functional adaptation of the fifth metatarsal bone in his left foot.

## Discussion and conclusions

A case of recurrent fifth metatarsal stress fractures with hypoparathyroidism in a professional soccer player was reported. In the present case, it was suspected that hypoparathyroidism was involved in the development of metatarsal stress re-fractures, in addition to the other multifactorial causes including screw fixation using small diameter screws or returning to play without an orthosis [18].

Previous reports showed that BMD is often above average in patients with hypoparathyroidism [2, 12, 16]. However, despite increased BMD, the risk of fragility fractures is higher in patients with hypoparathyroidism [5]. Furthermore, structural abnormalities of bone in such patients include increased cortical and trabecular width and cancellous bone volume, as well as markedly reduced bone turnover [14, 15].

In the present case, the patient had an abnormally high BMD. The patient might have had a structural abnormality of bone, as in the previous reports. Deepak et al. showed that there are many risk factors for stress fractures, but hypoparathyroidism was not included [13]. However, this is the first case of recurrent stress fractures with hypoparathyroidism. Thus, hypoparathyroidism could also be a risk factor for stress fractures. Furthermore, in the present case, the patient had no symptoms of hypoparathyroidism, and there are numerous asymptomatic patients with the disease [11]. Therefore, there might be other patients with hypoparathyroidism who develop stress fractures. We believe that serum calcium levels should be checked in patients with stress fractures, and if the serum calcium level is low, serum i-PTH levels also need to be checked, since hypoparathyroidism should be considered.

Regarding treatment, there are no formal guidelines for the management of hypoparathyroidism [6]. It is true that previous reports showed the effectiveness of teriparatide. However, standard therapy of hypoparathyroidism is said to be vitamin D supplementation with the goal of maintaining serum calcium within the low-normal range and avoiding hypercalciuria [16]. Therefore, the present patient was treated with alfacalcidol because he had no symptoms other than the fifth metatarsal stress fracture. In the present case, the serum calcium level increased immediately after the start of alfacalcidol, and the patient could return to sports without any problem.

In conclusion, this is the first reported case of recurrent fifth metatarsal stress fractures with hypoparathyroidism. Hypoparathyroidism might be one of the risk factors for stress fractures, and there are many asymptomatic patients with the disease. Therefore, there might be patients with undiagnosed hypoparathyroidism who develop stress fractures. We believe that serum calcium levels need to be checked in patients with stress fractures, and if the serum calcium is low, serum i-PTH levels also need to be checked.

## Data Availability

This is a case report of a single patient, to protect privacy and respect confidentiality; none of the raw data has been made available in any public repository. The original reports, laboratory studies, imaging studies and outpatient clinic records are retained as per normal procedure within the medical records of our institution.

## Abbreviations

i-PTH: intact parathyroid hormone
BMD: Bone mineral density
CT: Computed tomography
